# Bis[2-(thio­phen-2-yl)quinoxaline-κ*N* ^4^]silver(I) perchlorate

**DOI:** 10.1107/S2414314623002468

**Published:** 2023-03-21

**Authors:** Guy Crundwell

**Affiliations:** a Department of Chemistry & Biochemistry, Central Connecticut State University, 1619 Stanley Street, New Britain, CT 06053, USA; Goethe-Universität Frankfurt, Germany

**Keywords:** crystal structure, silver(I), quinoxalines, thienyl rings

## Abstract

The crystal of the title salt, [Ag(C_12_H_8_N_2_S)_2_]ClO_4_, has *C*2/*c* symmetry whereby the silver(I) atom sits on a twofold rotation axis, as does the perchlorate anion, which is disordered about this axis. The thienylquinoxaline ligand is nearly planar with the thienyl ring making a dihedral angle of 10.88 (8)° with respect to the quinoxaline moiety.

## Abstract 2

The structure of bis­[2-(thio­phen-2-yl)quinoxaline-κ*N* ^4^]silver(I) perchlorate, [Ag(C_12_H_8_N_2_S)_2_]ClO_4_, has a silver(I) metal center that sits on a twofold symmetry axis, as does a disordered perchlorate anion. The thienylquinoxaline ligand is nearly planar with the thienyl ring making a dihedral angle of 10.88 (8)° with respect to the quinoxaline moiety.

## Graphical abstract

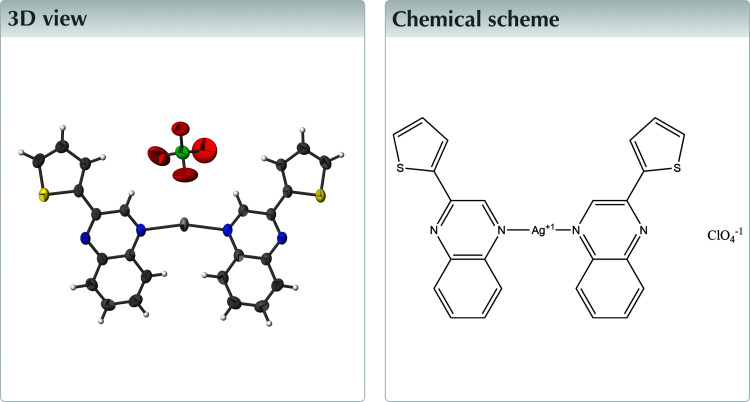

## Structure description

**Figure 1:**
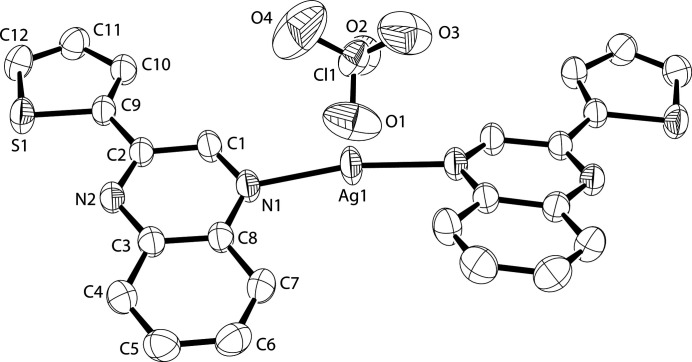
A view of the title compound (Farrugia, 2012 ▸). Displacement ellipsoids are drawn at the 50% probability level.

The silver(I) metal center sits on a twofold symmetry axis (Fig. 1 ▸). As a result of the position of the twofold axis, the two thienylquinoxaline ligands, which are bonding *via* their quinoxaline N atoms, adopt a configuration whereby both of the quinoxaline units are pointing to the same side of the molecule, as opposed to the tetra­fluorido­borate complex with the same cation, which crystallizes with the two ligands pointing in opposite directions (Crundwell, 2013 ▸). The thienylquinoxaline ligand is nearly planar, with the thienyl ring making a dihedral angle of 10.88 (8)° with respect to the quinoxaline moiety. This is similar to the nearly planar ligand configuration in the tetra­fluorido­borate salt (Crundwell, 2013 ▸).

## Synthesis and crystallization

Crystals were grown by combining warmed methano­lic solutions of AgClO_4_ and 2-thienylquinoxaline in a 1:2 molar ratio. The combined solution was pipetted into test tubes, which were then placed into amber vials and loosely sealed until small colorless crystals were observed. Crystals were harvested and used immediately since the silver salts deteriorate in light over days. When measuring of melting points was attempted, the crystals decomposed.

## Refinement

Crystal data, data collection and structure refinement details are summarized in Table 1 ▸. The perchlorate disorder was treated by suppressing the generation of additional solvent atoms due to the anion’s position on the symmetry axis. The perchlorate bond distances and oxygen-to-oxygen distances were restrained to 1.41 (1) and 2.30 (2) Å, respectively.

## Supplementary Material

Crystal structure: contains datablock(s) I. DOI: 10.1107/S2414314623002468/bt4134sup1.cif

Structure factors: contains datablock(s) I. DOI: 10.1107/S2414314623002468/bt4134Isup2.hkl

CCDC reference: 2248354

Additional supporting information:  crystallographic information; 3D view; checkCIF report

## Figures and Tables

**Table 1:** Experimental details

Crystal data	
Chemical formula	[Ag(C_12_H_8_N_2_S)_2_]ClO_4_
*M*_r_	631.85
Crystal system, space group	Monoclinic, *C*2/*c*
Temperature (K)	293
*a*, *b*, *c* (Å)	29.8739 (8), 10.6344 (4), 7.6425 (4)
β (°)	99.930 (4)
*V* (Å^3^)	2391.58 (17)
*Z*	4
Radiation type	Mo *K*α
μ (mm^−1^)	1.17
Crystal size (mm)	0.31 × 0.30 × 0.22

Data collection	
Diffractometer	Xcalibur CCD, Sapphire3
Absorption correction	Multi-scan (*CrysAlis PRO*; Agilent, 2014 ▸)
*T*_min_, *T*_max_	0.770, 1.000
No. of measured, independent and observed [*I* > 2σ(*I*)] reflections	13270, 3957, 2441
*R*_int_	0.027
(sin θ/λ)_max_ (Å^−1^)	0.753

Refinement	
*R*[*F* ^2^ > 2σ(*F* ^2^)], *wR*(*F* ^2^), *S*	0.030, 0.072, 0.82
No. of reflections	3957
No. of parameters	187
No. of restraints	10
H-atom treatment	H-atom parameters constrained
Δρ_max_, Δρ_min_ (e Å^−3^)	0.36, −0.29

Computer programs: *CrysAlis CCD* (Oxford Diffraction, 2009 ▸), *CrysAlis RED* (Oxford Diffraction, 2009 ▸), *SHELXS97* (Sheldrick, 2008 ▸), *SHELXL* (Sheldrick, 2015 ▸), and *OLEX2* (Dolomanov *et al.*, 2009 ▸).

## full crystallographic data

### Crystal data

**Table:**

[Ag(C_12_H_8_N_2_S)_2_]ClO_4_	*F*(000) = 1264
*M**_r_* = 631.85	*D*_x_ = 1.755 Mg m^−^^3^
Monoclinic, *C*2/*c*	Mo *K*α radiation, λ = 0.71073 Å
*a* = 29.8739 (8) Å	Cell parameters from 5344 reflections
*b* = 10.6344 (4) Å	θ = 4.1–32.4°
*c* = 7.6425 (4) Å	µ = 1.17 mm^−^^1^
β = 99.930 (4)°	*T* = 293 K
*V* = 2391.58 (17) Å^3^	Block, colorless
*Z* = 4	0.31 × 0.30 × 0.22 mm

### Data collection

**Table:**

Xcalibur CCD, Sapphire3 diffractometer	3957 independent reflections
Radiation source: fine-focus sealed tube	2441 reflections with *I* > 2σ(*I*)
Graphite monochromator	*R*_int_ = 0.027
ω scans	θ_max_ = 32.4°, θ_min_ = 4.2°
Absorption correction: multi-scan (CrysAlisPRO; Agilent, 2014)	*h* = −44→44
*T*_min_ = 0.770, *T*_max_ = 1.000	*k* = −15→15
13270 measured reflections	*l* = −11→10

### Refinement

**Table:**

Refinement on *F*^2^	Primary atom site location: structure-invariant direct methods
Least-squares matrix: full	Secondary atom site location: difference Fourier map
*R*[*F*^2^ > 2σ(*F*^2^)] = 0.030	Hydrogen site location: inferred from neighbouring sites
*wR*(*F*^2^) = 0.072	H-atom parameters constrained
*S* = 0.82	*w* = 1/[σ^2^(*F*_o_^2^) + (0.0428*P*)^2^] where *P* = (*F*_o_^2^ + 2*F*_c_^2^)/3
3957 reflections	(Δ/σ)_max_ < 0.001
187 parameters	Δρ_max_ = 0.36 e Å^−^^3^
10 restraints	Δρ_min_ = −0.29 e Å^−^^3^

### Special details

**Table:**

Experimental. Hydrogen atoms were included in calculated positions with a C-H distance of 0.93 Å and were included in the refinement in riding motion approximation with U_iso_ = 1.2U_eq_ of the carrier atom. Anion disorder treated with PART -1 and DFIX restraints.
Geometry. All esds (except the esd in the dihedral angle between two l.s. planes) are estimated using the full covariance matrix. The cell esds are taken into account individually in the estimation of esds in distances, angles and torsion angles; correlations between esds in cell parameters are only used when they are defined by crystal symmetry. An approximate (isotropic) treatment of cell esds is used for estimating esds involving l.s. planes.
Refinement. Refinement of F^2^ against ALL reflections. The weighted R-factor wR and goodness of fit S are based on F^2^, conventional R-factors R are based on F, with F set to zero for negative F^2^. The threshold expression of F^2^ > 2sigma(F^2^) is used only for calculating R-factors(gt) etc. and is not relevant to the choice of reflections for refinement. R-factors based on F^2^ are statistically about twice as large as those based on F, and R- factors based on ALL data will be even larger. Hydrogen atoms were placed in calculated positions with a C—H distance of 0.93 Å and were included in the refinement in a riding model approximation with *U_iso_* = 1.2 *U_eq_*(C). Difference maps and oblong thermal parameters indicated that the perchlorate anion was disordered about a twofold axis.

### Fractional atomic coordinates and isotropic or equivalent isotropic displacement parameters (Å^2^)

**Table:**

	*x*	*y*	*z*	*U*_iso_*/*U*_eq_	Occ. (<1)
Ag1	1.0000	0.15837 (2)	0.2500	0.05278 (10)	
S1	0.774221 (15)	0.02088 (5)	−0.17950 (8)	0.04958 (14)	
N1	0.92717 (5)	0.18494 (14)	0.1583 (2)	0.0404 (4)	
N2	0.83748 (4)	0.22291 (14)	−0.0167 (2)	0.0382 (4)	
C1	0.90225 (6)	0.09241 (18)	0.0828 (3)	0.0413 (4)	
H1	0.9148	0.0123	0.0854	0.050*	
C2	0.85637 (5)	0.11036 (17)	−0.0035 (3)	0.0353 (4)	
C3	0.86277 (6)	0.32029 (16)	0.0635 (3)	0.0372 (4)	
C4	0.84337 (7)	0.44149 (18)	0.0619 (3)	0.0458 (5)	
H4	0.8137	0.4548	0.0043	0.055*	
C5	0.86803 (8)	0.53904 (19)	0.1447 (3)	0.0503 (5)	
H5	0.8550	0.6186	0.1424	0.060*	
C6	0.91271 (8)	0.52096 (19)	0.2333 (3)	0.0514 (5)	
H6	0.9292	0.5887	0.2881	0.062*	
C7	0.93217 (6)	0.40506 (19)	0.2397 (3)	0.0472 (5)	
H7	0.9617	0.3935	0.3000	0.057*	
C8	0.90762 (5)	0.30265 (17)	0.1553 (3)	0.0366 (4)	
C9	0.82923 (6)	0.00112 (16)	−0.0714 (3)	0.0373 (4)	
C10	0.83990 (6)	−0.12423 (17)	−0.0520 (3)	0.0436 (5)	
H10	0.8681	−0.1542	0.0031	0.052*	
C11	0.80301 (7)	−0.2030 (2)	−0.1258 (3)	0.0509 (5)	
H11	0.8043	−0.2904	−0.1234	0.061*	
C12	0.76607 (7)	−0.1374 (2)	−0.1993 (3)	0.0514 (5)	
H12	0.7391	−0.1742	−0.2547	0.062*	
Cl1	0.9978 (2)	−0.17969 (10)	0.2283 (8)	0.0577 (9)	0.50
O1	1.00756 (19)	−0.0707 (4)	0.1315 (7)	0.1067 (16)	0.50
O2	1.00003 (13)	−0.2842 (3)	0.1151 (5)	0.0727 (10)	0.50
O3	0.95369 (19)	−0.1661 (5)	0.2619 (11)	0.124 (2)	0.50
O4	1.0300 (3)	−0.1931 (7)	0.3832 (9)	0.163 (3)	0.50

### Atomic displacement parameters (Å^2^)

**Table:**

	*U*^11^	*U*^22^	*U*^33^	*U*^12^	*U*^13^	*U*^23^
Ag1	0.02387 (9)	0.05375 (14)	0.07518 (19)	0.000	−0.00703 (9)	0.000
S1	0.0328 (2)	0.0521 (3)	0.0581 (4)	−0.00168 (19)	−0.0080 (2)	0.0023 (2)
N1	0.0255 (6)	0.0442 (9)	0.0494 (10)	0.0015 (5)	0.0002 (6)	0.0004 (7)
N2	0.0285 (6)	0.0427 (8)	0.0410 (10)	0.0017 (6)	−0.0006 (6)	0.0004 (7)
C1	0.0281 (7)	0.0404 (10)	0.0531 (13)	0.0023 (7)	0.0007 (8)	0.0003 (9)
C2	0.0274 (7)	0.0420 (9)	0.0355 (10)	−0.0003 (7)	0.0023 (7)	0.0003 (8)
C3	0.0325 (8)	0.0417 (10)	0.0366 (11)	0.0030 (7)	0.0036 (7)	0.0020 (8)
C4	0.0430 (10)	0.0450 (10)	0.0468 (13)	0.0074 (8)	0.0007 (9)	0.0023 (9)
C5	0.0588 (12)	0.0395 (10)	0.0521 (14)	0.0082 (9)	0.0080 (10)	−0.0001 (9)
C6	0.0549 (12)	0.0460 (11)	0.0529 (14)	−0.0096 (9)	0.0077 (10)	−0.0094 (10)
C7	0.0353 (9)	0.0502 (11)	0.0535 (14)	−0.0054 (8)	0.0003 (9)	−0.0055 (10)
C8	0.0283 (7)	0.0421 (9)	0.0389 (11)	−0.0002 (6)	0.0043 (7)	0.0004 (8)
C9	0.0273 (7)	0.0459 (10)	0.0374 (11)	−0.0007 (7)	0.0016 (7)	0.0006 (8)
C10	0.0342 (8)	0.0433 (10)	0.0510 (13)	−0.0003 (7)	0.0012 (8)	−0.0034 (9)
C11	0.0516 (11)	0.0433 (10)	0.0545 (14)	−0.0052 (9)	−0.0002 (10)	−0.0024 (10)
C12	0.0429 (9)	0.0547 (12)	0.0527 (14)	−0.0124 (9)	−0.0030 (9)	−0.0010 (10)
Cl1	0.0621 (12)	0.0518 (5)	0.053 (3)	0.0063 (9)	−0.0079 (17)	−0.0050 (8)
O1	0.152 (4)	0.055 (2)	0.132 (4)	−0.002 (3)	0.078 (4)	−0.005 (2)
O2	0.088 (2)	0.0528 (19)	0.074 (3)	0.0035 (19)	0.006 (2)	−0.0154 (18)
O3	0.116 (4)	0.105 (4)	0.172 (7)	0.036 (3)	0.082 (4)	0.035 (4)
O4	0.206 (8)	0.177 (7)	0.081 (4)	0.003 (6)	−0.045 (5)	−0.009 (4)

### Geometric parameters (Å, º)

**Table:**

Ag1—N1	2.1860 (13)	C5—C6	1.402 (3)
Ag1—N1^i^	2.1861 (13)	C6—H6	0.9300
S1—C9	1.7202 (17)	C6—C7	1.360 (3)
S1—C12	1.704 (2)	C7—H7	0.9300
N1—C1	1.307 (2)	C7—C8	1.406 (3)
N1—C8	1.380 (2)	C9—C10	1.373 (3)
N2—C2	1.320 (2)	C10—H10	0.9300
N2—C3	1.364 (2)	C10—C11	1.421 (3)
C1—H1	0.9300	C11—H11	0.9300
C1—C2	1.427 (2)	C11—C12	1.344 (3)
C2—C9	1.460 (2)	C12—H12	0.9300
C3—C4	1.412 (2)	Cl1—O1	1.432 (5)
C3—C8	1.414 (2)	Cl1—O2	1.417 (5)
C4—H4	0.9300	Cl1—O3	1.392 (7)
C4—C5	1.363 (3)	Cl1—O4	1.399 (6)
C5—H5	0.9300		
			
N1—Ag1—N1^i^	165.14 (8)	C6—C7—H7	120.0
C12—S1—C9	91.83 (9)	C6—C7—C8	119.98 (18)
C1—N1—Ag1	120.27 (12)	C8—C7—H7	120.0
C1—N1—C8	117.94 (14)	N1—C8—C3	119.39 (15)
C8—N1—Ag1	121.17 (11)	N1—C8—C7	120.63 (15)
C2—N2—C3	117.24 (13)	C7—C8—C3	119.98 (17)
N1—C1—H1	118.9	C2—C9—S1	119.97 (13)
N1—C1—C2	122.13 (16)	C10—C9—S1	110.81 (13)
C2—C1—H1	118.9	C10—C9—C2	129.05 (16)
N2—C2—C1	121.37 (16)	C9—C10—H10	123.8
N2—C2—C9	119.37 (14)	C9—C10—C11	112.34 (17)
C1—C2—C9	119.20 (16)	C11—C10—H10	123.8
N2—C3—C4	119.64 (15)	C10—C11—H11	123.7
N2—C3—C8	121.78 (15)	C12—C11—C10	112.58 (18)
C4—C3—C8	118.53 (16)	C12—C11—H11	123.7
C3—C4—H4	119.9	S1—C12—H12	123.8
C5—C4—C3	120.16 (17)	C11—C12—S1	112.43 (15)
C5—C4—H4	119.9	C11—C12—H12	123.8
C4—C5—H5	119.6	O2—Cl1—O1	106.5 (5)
C4—C5—C6	120.87 (19)	O3—Cl1—O1	107.2 (4)
C6—C5—H5	119.6	O3—Cl1—O2	109.9 (5)
C5—C6—H6	119.8	O3—Cl1—O4	112.9 (6)
C7—C6—C5	120.47 (18)	O4—Cl1—O1	110.4 (6)
C7—C6—H6	119.8	O4—Cl1—O2	109.8 (4)
			
Ag1—N1—C1—C2	−170.09 (15)	C2—N2—C3—C8	0.8 (3)
Ag1—N1—C8—C3	167.57 (14)	C2—C9—C10—C11	175.2 (2)
Ag1—N1—C8—C7	−11.3 (3)	C3—N2—C2—C1	−3.4 (3)
S1—C9—C10—C11	0.0 (3)	C3—N2—C2—C9	173.90 (18)
N1^i^—Ag1—N1—C1	162.84 (16)	C3—C4—C5—C6	−0.4 (3)
N1^i^—Ag1—N1—C8	−7.97 (15)	C4—C3—C8—N1	−179.96 (19)
N1—C1—C2—N2	2.6 (3)	C4—C3—C8—C7	−1.1 (3)
N1—C1—C2—C9	−174.71 (19)	C4—C5—C6—C7	−0.6 (4)
N2—C2—C9—S1	3.9 (3)	C5—C6—C7—C8	0.8 (4)
N2—C2—C9—C10	−170.9 (2)	C6—C7—C8—N1	179.0 (2)
N2—C3—C4—C5	178.7 (2)	C6—C7—C8—C3	0.1 (3)
N2—C3—C8—N1	2.6 (3)	C8—N1—C1—C2	1.0 (3)
N2—C3—C8—C7	−178.51 (19)	C8—C3—C4—C5	1.2 (3)
C1—N1—C8—C3	−3.5 (3)	C9—S1—C12—C11	0.7 (2)
C1—N1—C8—C7	177.7 (2)	C9—C10—C11—C12	0.5 (3)
C1—C2—C9—S1	−178.79 (16)	C10—C11—C12—S1	−0.8 (3)
C1—C2—C9—C10	6.4 (4)	C12—S1—C9—C2	−176.09 (18)
C2—N2—C3—C4	−176.53 (19)	C12—S1—C9—C10	−0.42 (18)

Symmetry code: (i) −*x*+2, *y*, −*z*+1/2.
